# Effects of Dietary Probiotic (*Bacillus subtilis*) Supplementation on Carcass Traits, Meat Quality, Amino Acid, and Fatty Acid Profile of Broiler Chickens

**DOI:** 10.3389/fvets.2021.767802

**Published:** 2021-11-22

**Authors:** Xiaopeng Tang, Xuguang Liu, Hu Liu

**Affiliations:** ^1^State Engineering Technology Institute for Karst Desertfication Control, School of Karet Science, Guizhou Normal University, Guiyang, China; ^2^State Key Laboratory of Grassland Agro-Ecosystems, School of Life Sciences, Lanzhou University, Lanzhou, China

**Keywords:** *Bacillus subtilis*, broiler, carcass trait, meat quality, amino acid, fatty acid

## Abstract

The aim of the present study was to evaluate the effects of dietary supplementation with or without *Bacillus subtilis* (*B. subtilis*) on carcass traits, meat quality, amino acids, and fatty acids of broiler chickens. In total, 160 1-day-old *Arbor Acres* male broiler chicks were divided into two groups with eight replicates of 10 chicks each. Chickens received basal diets without (CN group) or with 500 mg/kg *B. subtilis* (BS group) for 42 days. Eight chickens from each group were slaughtered at the end of the trial, and carcass traits, meat quality, chemical composition, amino acid, and fatty acid profile of meat were measured. The results showed that the breast muscle (%) was higher in BS than in CN (*p* < 0.05), while abdominal fat decreased (*p* < 0.05). The pH_24h_ of thigh muscle was increased (*p* < 0.05) when supplemented with BS; however, drip loss, cooking loss of breast muscle, and shear force of thigh muscle decreased (*p* < 0.05). Lysine (Lys), methionine (Met), glutamic acid (Glu), and total essential amino acid (EAA) in breast muscle and Glu in thigh muscle were greater in BS than in CN (*p* < 0.05). C16:1, C18:1n9c, and MUFA in breast muscle and thigh muscle were greater in BS than in CN (*p* < 0.05). In conclusion, dietary supplementation with *B. subtilis* could improve the carcass traits and meat quality of broilers, which is beneficial for the consumers due to the improved fatty acid profile and amino acid composition.

## Introduction

The widespread use of antibiotics has led to the emergence of resistant bacteria and drug residues in animal products, which directly or indirectly endangers human's health and environmental safety (1, 2). Nowadays, with the development of molecular biology, genetic improvements in broiler production have improved body size and growth rate of broilers, resulting in a higher yield of meat in broilers (3). However, the meat quality was decreased drastically with the rapid growth of broilers. Thus, in order to satisfy people's growing demand for high-quality meat, the improvement of meat quality becomes a current hot topic in the field of animal nutrition. Previous studies have demonstrated that probiotics can play a role in improving the microbial balance and intestinal environment (4–7) and are believed to promote the growth performance (8) and meat quality of broilers by multiple ways (9, 10). It is important to note that in all of these studies, *Bacillus* species, including *Bacillus subtilis* (*B. subtilis*), *Bacillus cereus* (*B. cereus*), *Bacillus clausii* (*B. clausii*), and *Bacillus coagulans* (*B. coagulans*), have been identified as effective probiotics to promote animal growth, maintain intestinal barrier function, and promote meat quality of broilers (9, 11–16).

The adverse environment such as low pH value, high temperature, and relatively greater bile salt within gastrointestinal tract is a severe challenge for probiotics survival (17). *B. subtilis* is a spore gram-positive aerobic bacterium that has a better heat stability and higher acid tolerance, making it a potential feed additive in animal production (18, 19). Previous studies have demonstrated that the properties of probiotic bacteria vary from strain to strain (20). With respect to *B. subtilis*, multiple strains, including *B. subtilis* DSM 17299 (19), *B. subtilis* fmbJ (12), *B. subtili*s RX7 and B2A (13), *B. subtilis* C-3102 (21), *B. subtilis* 29784 (22), *B. subtilis* BYS2 and BG5 (23), *B. subtilis* 1781 and 747 (24), and *B. subtilis* DSM 32315 (25–28), have been gaining interest as they have been indicated to improve the growth performance, feed efficiency, antioxidant capability, immune function, and intestinal microflora balance of broilers. *B. subtilis* DSM 32315, a unique naturally occurring strain, has shown to improve growth performance and intestinal microflora balance of broilers (25–29). For example, Ma et al. (25) showed that dietary supplementation with *B. subtilis* DSM 32315 improved growth performance, intestinal structure, and the manipulation of cecal microbial composition of broilers. Sokale et al. (26) indicated that dietary supplementation with *B. subtilis* DSM 32315 can maintain the intestinal structural integrity and ameliorate the pathology and performance detriments associated with necrotic enteritis challenge. However, previous studies about the application of *B. subtilis* DSM 32315 in poultry mainly concentrated in growth performance and intestinal health; the effect of dietary supplementation with *B. subtilis* DSM 32315 on carcass traits and meat quality of broiler chickens has not been investigated in detail. Thus, the present study aims to investigate the effects of *B. subtilis* DSM 32315 supplementation on carcass traits, meat quality, amino acid (AA), and fatty acid profile of broiler chickens to provide more references for the application of *B. subtilis* in poultry production.

## Materials and Methods

### Birds, Experimental Design, Diets, and Management

The experimental procedures involving animals were reviewed and approved by the animal welfare committee of the Guizhou Normal University (Guiyang, China). A total of 160 1-day-old Arbor Acres (AA) male broiler chicks with similar initial body weights were randomly divided into two groups with eight replicates of 10 chicks each. Chickens received basal diets without (CN group) or with 500 mg/kg *B. subtilis* (BS group, *B. subtilis* DSM 32315, 2.0 × 10^9^ spores/g, Evonik Nutrition & Care GmbH, Germany) throughout the trial period (25). The basal diets (Table 1) were formulated according to the nutrient requirements of the Chinese Feeding Standard of Chicken (NY/T 33-2004, China's Ministry of Agriculture). The experiment lasted for 42 days, which was divided into two phases (phase 1: days 1–21 and phase 2: days 22–42). All birds were raised in wire cages in an environmentally controlled room with continuous incandescent white light throughout the experiment and had *ad libitum* access to water and feed. The room temperature was maintained at 33°C for the first 3 days and at 32°C to 30°C for days 4–7, and then reduced by 23°C per week until it reached 22–24°C.

**Table 1:** Composition and nutrient level of basal diet (as feed basis).

**Ingredients**	**1–21 days**	**22–42 days**
Corn	58.52	61.80
Soybean meal	34.45	30.77
Soybean oil	3.15	3.97
CaHPO_4_	1.63	1.30
Limestone	0.90	0.91
NaCl	0.35	0.35
*DL*-Met	0.18	0.10
*L*-Lys·HCl	0.02	0.00
Vitamin premix^a^	0.10	0.10
Mineral premix^b^	0.25	0.25
Choline chloride	0.20	0.20
Zeolite powder	0.25	0.25
Total	100	100
**Nutrient levels^c^**		
ME (MJ/kg)	12.51	12.87
CP (%)	21.48	19.99
Ca (%)	1.00	0.90
Available phosphorus (%)	0.45	0.40
Total phosphorus (%)	0.68	
Lysine (%)	1.15	1.00
Methionine (%)	0.50	0.40
Methionine + cystine (%)	0.92	0.78
Threonine (%)	0.81	0.75

a *Vitamin premix provided the following per kilogram of diets: VA 12,000 IU, VD_3_ 2,500 IU, VE 30 IU, VK_3_ 2.65 mg, VB_1_ 2 mg, VB_2_ 6 mg, VB_3_ 10 mg, VB_12_ 0.025 mg, biotin 0.12 mg, folic acid 1.25 mg, pantothenic acid 12 mg, nicotinic acid 50 mg*.

b *Mineral premix provided the following per kilogram of diets: Cu (as copper sulfate) 8 mg, Zn (as zinc sulfate) 75 mg, Fe (as ferrous sulfate) 80 mg, Mn (as manganese sulfate) 100 mg, Se (as sodium selenite) 0.15 mg, and I (as potassium iodide) 0.35 mg*.

c *Nutrition levels were calculated values*.

### Carcass Traits

At the end of the feeding trial, the diet was removed 12 h before slaughter. Eight middle-weighted birds (one bird/replicate) in each treatment were selected for sample collection. All selected animals were weighed individually, and then slaughtered by severing the jugular vein after electroshock. Then, the carcasses were cut according to a standardized procedure (NY/T 823-2004) to determine the dressing percentage, semi-eviscerated percentage (%), eviscerated percentage (%), breast muscle (%), and abdominal fat (%). After defeathering, head removal, hock cut, and evisceration, the carcass weight, semi-eviscerated weight, and eviscerated weight were recorded. The dressing percentage (%) was calculated by dividing final body weight with carcass weight [(carcass weight/body weight) × 100], the semi-eviscerated percentage (%) was calculated by dividing final body weight with semi-eviscerated weight [(semi-eviscerated weight/body weight) × 100], and the eviscerated percentage (%) was calculated by dividing final body weight with eviscerated weight [(eviscerated weight/body weight) × 100]. The breast muscle, leg muscle, and abdominal fat were removed and weighed. The breast muscle (%) was calculated by dividing eviscerated weight with breast muscle weight [(breast muscle weight/eviscerated weight) × 100], the leg muscle (%) was calculated by dividing eviscerated weight with leg muscle weight [(leg muscle weight/eviscerated weight) × 100], and the abdominal fat (%) was calculated by dividing eviscerated weight plus abdominal fat weight with abdominal fat weight [abdominal fat weight/(eviscerated weight + abdominal fat weight) × 100]. Approximately 5.0 g of muscles from the medial pectoralis major and thigh were sampled for chemical composition measurement. The remaining muscles from each bird were kept at 4°C for the analysis of meat quality.

### Meat Quality Assessment

Meat quality was assessed by determining pH, meat color, drip loss, shear force (N), and cooking loss of the breast and thigh muscle. The pH of breast and thigh muscle was measured at 24 h (pH_24h_) postmortem by insertion of a handheld pH meter (Russell CD700, Russell pH Limited, Germany). Meat color, including lightness (L^*^), redness (a^*^), and yellowness (b^*^), was measured using Chroma Meter (Opto-Star Lab, Matthaus, Germany) according to the manufacturer's instructions. The measurements of drip loss from 0 to 24 h were conducted as described by Zhou et al. (16). Briefly, ~30 g of muscle sample was weighed and put into zip-lock plastic bags and stored in a chilling room at 4°C for 24 h and then reweighed after wiping out the surface moisture, and the drip loss was calculated as: drip loss (%) = [(initial weight – final weight)/final weight] × 100. The measurements of shear force and cooking loss were conducted as described by Cai et al. (3) with appropriate modification. Approximately 20 g of a sample of muscle sample was weighted and heated in a water bath to a final internal temperature of 75°C. After cooling to room temperature, the muscle sample was reweighed after wiping out the surface moisture and the cooking loss was calculated as: cooking loss (%) = [(initial weight – final weight)/final weight] × 100. The sample used for the measurement of cooking loss was then used for the measurement of shear force. The sample was cut into 2 cm (length) × 1 cm (width) × 0.5 cm (height) along the direction of the muscle fibers, and the shear force was measured using a digital texture analyzer (Model 2000D, G-R, US).

### Chemical Composition Analysis

Muscle samples were cut into slices, dried in a vacuum-freeze dryer, and then ground into powder. The moisture, crude ash, crude protein (CP), and extracted fat (EE) were measured according to the methods described by AOAC [2000; (30)].

To measure the AA content in breast and thigh muscle, the samples were pretreated according to AOAC [2000; (30)]. Briefly, ~200 mg of fresh muscle sample was accurately weighed into the hydrolysis tube (about 15 ml), 10 ml of hydrochloric acid (6 mol/L) was added, and the mixed sample was filled with nitrogen, sealed, and put in a constant temperature drying box at 110°C for hydrolysis for 24 h; after cooling and mixing well, 100 μl of mixed sample was taken out and dried at 55°C and then 1,000 μl of AA sample diluent was added and mixed well; 1 ml of mixed sample was filtered used a needle filter (diameter 0.2 μm) and then analyzed using an automatic AA analyzer (L-8800; Hitachi, Tokyo, Japan) according to the manufacturer's instructions, and the contents of AA were expressed as mg/g wet tissue.

To measure the fatty-acid profiles in breast and thigh muscle, the samples were pretreated according to AOAC [2000; (30)]. Briefly, ~50–200 mg of freeze-dried powder samples was accurately weighed into a 10-ml glass tube, and 3 ml of chloroform–methanol–water (volume ratio: 8:4:3) mixture was added. The mixture was shaken in a whirlpool for 1 min, followed by ultrasound for 5 min in an ice bath, repeated three to five times, left for 2 h, and centrifuged at 1,500 rpm for 15 min. The chloroform layer was transferred to another 10-ml glass centrifuge tube and dried in a vacuum drying oven to obtain the mixture of fatty acid and glyceryl ester; 2 ml of KOH-CH_3_OH (0.5 mol/L) was added and placed in a slightly boiling water bath for 10 min; after cooling for 3 min, 2 ml of BF_3_-CH_3_OH solution (10%) was added and then placed in a water bath at 80°C to heat reflux for 2 min, cooled to room temperature, added 1 ml n-hexane and 3 ml of saturated NaCl solution, fully mixed, and centrifuged at room temperature at 2,000 rpm for 5 min, and then the upper n-hexane layer was absorbed into the gas phase vial for the fatty-acid profiles analyzed using an automatic fatty acid analyzer (Shimadzu GC-2010 plus, Japan) according to the manufacturer's instructions.

### Statistical Analysis

All the data were subjected to the paired *t-*test using SPSS 21.0 programs (SPSS, Inc., Chicago, IL, USA). Results are expressed as the mean ± standard deviation (SD). Probability values < 0.05 were taken to indicate statistical significance.

## Results

### Carcass Traits

The effects of *B. subtilis* supplementation on carcass traits of broiler chickens are presented in Table 2. Compared to the control group, diet supplementation with *B. subtilis* improved the breast muscle percentage (27.14 vs. 25.04%; *p* < 0.05), but decreased the abdominal fat percentage (1.52 vs. 1.74%; *p* < 0.05). No influences were observed in carcass weight, dressing percentage, semi-eviscerated percentage, eviscerated percentage, and leg muscle percentage (*P* > 0.10) among the treatments.

**Table 2:** Effects of *Bacillus subtilis* supplementation on carcass traits of broiler chickens.

**Item**	**CN**	**BS**	***p*-value**
Final body weight (g)	2,531.90 ± 130.28	2,568.92 ± 141.50	0.595
Carcass weight (g)	2,265.75 ± 69.53	2,320.53 ± 121.27	0.286
Dressing percentage (%)	89.58 ± 2.23	90.36 ± 2.05	0.477
Semi-eviscerated percentage (%)	81.80 ± 4.10	82.13 ± 4.49	0.877
Eviscerated percentage (%)	70.17 ± 2.14	71.40 ± 0.64	0.342
Breast muscle (%)	25.04 ± 1.60	27.14 ± 1.82	0.028
Leg muscle (%)	19.76 ± 1.46	20.01 ± 2.01	0.779
Abdominal fat (%)	1.74 ± 0.15	1.52 ± 0.18	0.017

*Values are expressed as means ± SD, n = 8; CN, Control group, chickens received a basal diet; BS, Bacillus subtilis group, chickens received a basal diet supplemented with Bacillus subtilis; p < 0.05 were taken to indicate statistical significance*.

### Meat Quality

The effects of *B. subtilis* supplementation on meat quality of broiler chickens are presented in Table 3. Supplementation with *B. subtilis* improved the pH_24h_ value (5.97 vs. 5.73; *p* < 0.05), but decreased the shear force (15.19 vs. 18.04; *p* < 0.05) in the thigh muscle compared to the control group. For the breast muscle, dietary supplementation with *B. subtilis* significantly increased Yellowness (b^*^) (7.89 vs. 7.61; *p* < 0.05) compared to the control group, but decreased the drip loss (3.24 vs. 4.08; *p* < 0.05) and the cooking loss (16.66 vs. 18.27; *p* < 0.05) compared to the control group. In addition, dietary supplementation with *B. subtilis* had a tendency to decrease shear force (19.81 vs. 24.67; *p* = 0.072) in the breast muscle compared to the control group. It seems that the thigh muscle had a lower Lightness (L^*^), Yellowness (b^*^), drip loss, cooking loss, and shear force (*p* < 0.05), but had a higher Redness (a^*^) (*P* < 0.05) than breast muscle.

**Table 3:** Effects of *Bacillus subtilis* supplementation on meat quality of broiler chickens.

**Item**	**Muscle**	**CN**	**BS**	***p*-value^#^**	***p*-value^‡^**
pH_24h_	Breast	5.71 ± 0.26	5.87 ± 0.22	0.204	0.479
	Thigh	5.73 ± 0.10	5.97 ± 0.24	0.023	
Lightness (L^*^)	Breast	46.19 ± 4.48	48.10 ± 3.94	0.381	<0.001
	Thigh	37.92 ± 3.26	40.96 ± 5.13	0.178	
Redness (a^*^)	Breast	5.22 ± 1.46	6.20 ± 1.69	0.231	<0.001
	Thigh	10.08 ± 4.61	12.73 ± 2.57	0.178	
Yellowness (b^*^)	Breast	7.61 ± 0.86	7.89 ± 1.31	0.036	<0.001
	Thigh	5.74 ± 1.56	6.38 ± 1.17	0.370	
Drip loss (%)	Breast	4.08 ± 0.74	3.24 ± 0.68	0.017	0.001
	Thigh	2.91 ± 0.61	2.53 ± 0.66	0.251	
Cooking loss (%)	Breast	18.27 ± 0.73	16.66 ± 1.35	0.010	<0.001
	Thigh	13.61 ± 2.44	12.74 ± 2.43	0.484	
Shear force (N)	Breast	24.67 ± 6.61	19.81 ± 2.49	0.072	0.001
	Thigh	18.04 ± 2.84	15.19 ± 1.66	0.028	

*Values are expressed as means ± SD, n = 8; CN, Control group, chickens received a basal diet; BS, Bacillus subtilis group, chickens received a basal diet supplemented with Bacillus subtilis;*

# *difference between CN group and BS group;*

‡ *difference between breast muscle and thigh muscle; p < 0.05 were taken to indicate statistical significance*.

### Meat Chemical Composition

The effects of *B. subtilis* supplementation on meat chemical composition (moisture, crude protein, ether extract, and crude ash) of broiler chickens are presented in Table 4. Dietary supplementation with *B. subtilis* had no effect on chemical composition of both breast and thigh muscles of broilers, and only had a tendency (*p* = 0.093) to increase ether extract content in the thigh muscle. In addition, the content of crude protein in breast muscle was higher, and ether extract was much lower than thigh muscle (*P* < 0.05).

**Table 4:** Effects of *Bacillus subtilis* supplementation on conventional chemical composition of broiler chickens.

**Item**	**Muscle**	**CN**	**BS**	***p*-value^#^**	***p*-value^‡^**
Moisture (%)	Breast	73.14 ± 3.55	72.22 ± 3.85	0.628	0.249
	Thigh	71.09 ± 2.51	70.73 ± 6.58	0.888	
Crude protein (%)	Breast	22.31 ± 2.41	24.37 ± 2.72	0.132	<0.001
	Thigh	19.23 ± 2.22	20.35 ± 2.40	0.351	
Ether extract (%)	Breast	1.50 ± 0.27	1.71 ± 0.22	0.120	<0.001
	Thigh	5.39 ± 0.70	6.01 ± 0.67	0.093	
Crude ash (%)	Breast	1.28 ± 0.59	1.27 ± 0.28	0.975	0.197
	Thigh	1.13 ± 0.38	1.08 ± 0.15	0.739	

*Values are expressed as means ± SD, n = 8; CN, Control group, chickens received a basal diet; BS, Bacillus subtilis group, chickens received a basal diet supplemented with Bacillus subtilis;*

# *difference between CN group and BS group;*

‡ *difference between breast muscle and thigh muscle; p < 0.05 were taken to indicate statistical significance tendency*.

### Meat Amino Acid Composition

The AA contents in breast muscle and thigh muscle are presented in Table 5. In the breast muscle, the content of lysine (Lys, 20.26 vs. 18.53 mg/g; *p* < 0.05), methionine (Met, 4.28 vs. 3.74 mg/100 g; *p* < 0.05), glutamic acid (Glu, 18.03 vs. 16.88 mg/100 g; *p* < 0.05), and the total essential amino acid (EAA, 81.19 vs. 77.20 mg/100 g; *p* < 0.05) was greater when supplemented with *B. subtilis* than the control group. In addition, dietary supplementation with *B. subtilis* had a tendency to increase the total AA (164.32 vs. 155.44 mg/100 g; *p* = 0.073) content in the breast muscle compared to the control group. In the thigh muscle, dietary *B. subtilis* supplementation increased the Glu (16.83 vs. 15.78 mg/100 g; *p* < 0.05) content and had a tendency to increase the Lys content (17.98 vs. 16.70 mg/100 g; *p* = 0.078) compared to the control group. It seems that most AAs [except phenylalanine (Phe), cysteine (Cys), glycine (Gly), and proline (Pro)] in the breast muscle were much higher than in the thigh muscle (*p* < 0.05).

**Table 5:** Effects of *Bacillus subtilis* supplementation on amino acid content in breast muscle and thigh muscle of broiler chickens (mg/100 g DM).

**Amino acids**	**Breast muscle**			**Thigh muscle**			***p*-value^‡^**
	**CN**	**BS**	***p*-value^#^**	**CN**	**BS**	***p*-value^†^**	
**Essential amino acid (EAA)**							
Threonine (Thr)	7.45 ± 0.54	7.88 ± 0.48	0.112	6.70 ± 0.42	6.86 ± 0.66	0.578	<0.001
Valine (Val)	6.78 ± 0.45	6.95 ± 0.33	0.402	5.87 ± 0.50	6.12 ± 0.60	0.385	<0.001
Methionine (Met)	3.74 ± 0.35	4.28 ± 0.50	0.025	3.45 ± 0.52	3.60 ± 0.44	0.528	0.009
Isoleucine (Ile)	7.29 ±0.69	7.42 ± 0.54	0.685	6.34 ± 0.59	6.56 ± 0.65	0.494	<0.001
Leucine (Leu)	9.80 ± 0.70	10.37 ± 0.63	0.107	8.80 ± 0.63	9.05 ± 0.88	0.517	<0.001
Histidine (His)	8.72 ± 0.98	8.85 ± 1.18	0.816	10.62 ± 1.29	11.46 ± 3.44	0.529	0.003
Phenylalanine (Phe)	4.33 ± 0.56	4.07 ± 0.29	0.263	3.84 ± 0.95	3.96 ±0.95	0.807	0.253
Arginine (Arg)	10.56 ± 0.77	11.09 ± 1.00	0.253	9.40 ± 0.60	9.70 ± 0.66	0.368	<0.001
Lysine (Lys)	18.53 ± 1.29	20.26 ± 1.05	0.011	16.70 ± 1.18	17.98 ± 1.49	0.078	<0.001
**Non-essential amino acid (NEAA)**							
Alanine (Ala)	12.56 ± 1.85	12.65 ± 2.65	0.736	10.71 ± 1.08	11.22 ± 1.22	0.393	0.007
Cysteine (Cys)	7.63 ± 0.48	8.46 ± 1.34	0.121	7.34 ± 0.73	7.84 ± 0.79	0.215	0.178
Aspartic acid (Asp)	14.26 ± 1.02	14.58 ± 0.61	0.472	12.39 ± 0.89	12.79 ± 1.22	0.469	<0.001
Serine (Ser)	5.10 ± 0.30	5.21 ± 0.33	0.518	4.70 ± 0.28	4.80 ± 0.37	0.550	0.001
Glutamic acid (Glu)	16.88 ± 1.21	18.03 ± 0.86	0.046	15.78 ± 0.87	16.83 ± 1.05	0.047	0.007
Glycine (Gly)	9.47 ± 0.72	10.80 ± 2.47	0.166	9.34 ± 1.55	10.13 ± 1.65	0.343	0.523
Tyrosine (Tyr)	4.93 ± 0.44	4.52 ± 0.49	0.105	4.25 ± 0.0.51	4.42 ± 0.57	0.546	0.039
Proline (Pro)	7.39 ± 0.47	8.58 ± 3.12	0.306	7.09 ± 0.85	7.45 ± 0.76	0.389	0.239
Total EAA	77.20 ± 2.46	81.19 ±3.74	0.025	72.20 ± 5.88	74.84 ± 9.07	0.501	0.009
Total NEAA	78.24 ± 4.08	83.13 ± 8.45	0.162	71.94 ±6.20	75.11 ± 6.31	0.328	0.007
Total AA	155.44 ± 5.78	164.32 ± 11.62	0.073	144.14 ± 4.05	149.95 ± 5.17	0.391	0.004
FAA	63.74 ± 3.89	67.46 ± 5.32	0.133	57.97 ± 4.77	60.31 ± 4.96	0.353	0.001

*Values are expressed as means ± SD, n = 8; CN, Control group, chickens received a basal diet; BS, Bacillus subtilis group, chickens received a basal diet supplemented with Bacillus subtilis; AA, amino acids; FAA (flavor amino acid), the sum of Glu, Asp, Gly, Arg, and Ala;*

# *difference between CN group and BS group in breast muscle;*

† *difference between CN group and BS group in thigh muscle;*

‡ *difference between breast muscle and thigh muscle; p < 0.05 were taken to indicate statistical significance*.

### Meat Fatty Acid Composition

The fatty acid contents in breast muscle and thigh muscle are presented in Table 6. The palmitoleic acid (C16:1, 3.228 vs. 1.938%; *p* < 0.05), oleic acid (C18:1n9c, 36.671 vs. 33.904%; *p* < 0.05), *Cis*-8,11,14-eicosatrienoate (C20:3n6, 1.387 vs. 1.109%; *p* < 0.05), and total monounsaturated fatty acid (MUFA, 41.190 vs. 37.074%; *p* < 0.05) were higher when supplemented with *B. subtilis*, but the palmitic acid (C16:0, 26.762 vs. 29.049%; *p* < 0.05) and arachidic acid (C20:0, 0.256 vs. 0.437%; *p* < 0.05) decreased in the breast muscle. In addition, dietary supplementation with *B. subtilis* had a tendency to increase eicosadienoic acid (C20:2, 0.468 vs. 0.569%) and the total unsaturated fatty acids (UFA, 59.160 vs. 55.514%, *p* = 0.083), and had a tendency to decrease the total saturated fatty acid (SFA, 40. 842 vs. 44.486%, *p* = 0.059) in the breast muscle compared to the control group. For the thigh muscle, dietary supplementation with *B. subtilis* increased the C16:1 (4.972 vs. 3.874%; *p* < 0.05), C18:1n9c (36.116 vs. 33.261%; *p* < 0.05), and MUFA (43.062 vs. 38.880%; *p* < 0.05), and decreased the C16:0 (23.747 vs. 24.924%; *p* < 0.05) and the total SFA (37.061 vs. 40.247%; *p* < 0.05) compared to the control group. There is a tendency to increase the myristoleic acid (C14:1, 0.060 vs. 0.054%, *p* = 0.057), nervonic acid (C24:1n9, 0.052 vs. 0.032%, *p* = 0.076), and the total UFA (62.939 vs. 59.752%, *p* = 0.066) in the thigh muscle when supplemented with *B. subtilis*. The results from the present study showed that the fatty acid content in breast muscle and thigh muscle is greatly different.

**Table 6:** Effects of *Bacillus subtilis* supplementation on fatty acids content in breast muscle and thigh muscle of broiler chickens (%).

**Fatty acids**	**Breast muscle**			**thigh muscle**			***p*-value^‡^**
	**CN**	**BS**	***p*-value^#^**	**CN**	**BS**	***p*-value^†^**	
**Saturated fatty acid (SFA)**							
C12:0	0.048 ± 0.017	0.040 ± 0.017	0.387	0.054 ± 0.011	0.047 ± 0.022	0.489	0.262
C14:0	0.432 ± 0.065	0.472 ± 0.080	0.290	0.602 ± 0.156	0.535 ± 0.053	0.267	0.002
C15:0	0.114 ± 0.014	0.104 ± 0.013	0.290	0.107 ± 0.018	0.087 ± 0.015	0.290	0.068
C16:0	29.049 ± 1.449	26.762 ± 2.031	0.021	24.924 ± 1.317	23.747 ± 0.798	0.049	<0.001
C17:0	0.340 ± 0.090	0.285 ± 0.063	0.179	0.326 ± 0.075	0.254 ± 0.104	0.132	0.476
C18:0	9.350 ± 0.610	8.855 ± 1.511	0.405	10.231 ± 2.630	8.939 ± 2.064	0.293	0.470
C20:0	0.437 ± 0.179	0.256 ± 0.155	0.048	0.204 ± 0.225	0.174 ± 0.124	0.746	0.020
C21:0	0.364 ± 0.171	0.385 ± 0.317	0.870	0.241 ± 0.142	0.325 ± 0.155	0.278	0.215
C22:0	0.170 ± 0.020	0.207 ± 0.110	0.360	0.107 ± 0.028	0.122 ± 0.052	0.483	0.002
C23:0	4.132 ± 0.692	3.433 ± 1.373	0.220	3.400 ± 1.042	2.794 ± 0.508	0.161	0.059
C24:0	0.017 ± 0.009	0.017 ± 0.007	0.988	0.017 ± 0.010	0.012 ± 0.005	0.233	0.381
**Unsaturated fatty acids (UFA)**							
C14:1	0.050 ± 0.029	0.085 ± 0.053	0.123	0.112 ± 0.054	0.171 ± 0.060	0.057	0.001
C16:1	1.938 ± 0.396	3.228 ± 1.391	0.024	3.874 ± 0.634	4.972 ± 0.851	0.011	<0.001
C17:1	0.039 ± 0.003	0.046 ± 0.013	0.138	0.064 ± 0.024	0.076 ± 0.031	0.378	0.001
C18:1n9t	0.769 ± 0.218	0.759 ± 0.178	0.921	1.051 ± 0.305	1.174 ± 0.531	0.581	0.005
C18:1n9c	33.904 ± 1.550	36.671 ± 2.556	0.020	33.261 ± 2.324	36.116 ± 2.402	0.030	0.521
C18:2n6t	0.075 ± 0.029	0.076 ± 0.019	0.921	0.037 ± 0.038	0.040 ± 0.051	0.914	0.006
C18:2n6c	11.797 ± 1.056	10.977 ± 2.223	0.362	14.835 ± 2.330	13.750 ± 1.227	0.263	<0.001
C18:3n3	0.881 ± 0.113	0.965 ± 0.263	0.422	1.542 ± 0.439	1.603 ± 0.431	0.782	<0.001
C18:3n6	0.219 ± 0.023	0.242 ± 0.051	0.253	0.301 ± 0.061	0.287 ± 0.122	0.781	0.018
C20:1n9	0.3160.037 ±	0.350 ± 0.066	0.323	0.439 ± 0.092	0.444 ± 0.117	0.926	0.001
C20:2	0.569 ± 0.060	0.468 ± 0.130	0.065	0.426 ± 0.070	0.370 ± 0.138	0.322	0.004
C20:3n6	1.109 ± 0.158	1.387 ± 0.247	0.018	0.562 ± 0.095	0.568 ± 0.228	0.955	<0.001
C20:3n3	0.065 ± 0.016	0.052 ± 0.020	0.196	0.051 ± 0.037	0.053 ± 0.044	0.952	0.534
C20:5N3	0.392 ± 0.082	0.386 ± 0.184	0.931	0.129 ± 0.035	0.152 ± 0.102	0.544	<0.001
C22:1n9	0.055 ± 0.013	0.046 ± 0.015	0.235	0.046 ± 0.027	0.056 ± 0.031	0.502	0.938
C22:2	0.015 ± 0.008	0.020 ± 0.008	0.207	0.022 ± 0.013	0.029 ± 0.011	0.318	0.031
C22:6n3	3.317 ± 0.553	3.395 ± 1.040	0.855	2.965 ± 1.086	3.024 ± 0.631	0.897	0.228
C24:1n9	0.004 ± 0.005	0.005 ± 0.005	0.642	0.032 ± 0.022	0.052 ± 0.020	0.076	<0.001
SFA	44.486 ± 2.141	40.842 ± 4.535	0.059	40.247 ± 3.395	37.061 ± 2.171	0.042	0.003
MUFA	37.074 ± 1.842	41.190 ± 3.955	0.018	38.880 ± 2.595	43.062 ± 3.099	0.011	0.157
PUFA	18.440 ± 1.242	17.970 ± 2.277	0.616	20.872 ± 2.988	19.876 ± 2.288	0.466	0.010
UFA	55.514 ± 1.666	59.160 ± 5.274	0.083	59.752 ± 4.178	62.939 ± 1.727	0.066	0.007

*Values are expressed as means ± SD, n = 8; CN, Control group, chickens received a basal diet; BS, Bacillus subtilis group, chickens received a basal diet supplemented with Bacillus subtilis; C12:0, Lauric acid; C14:0, Myristic acid; C15:0, Pentadecanoic acid; C16:0, Palmitic acid; C17:0, Heptadecanoic acid; C18:0, Stearic acid; C20:0, Arachidic acid; C21:0, Heneicosanoic acid; C22:0, Docosanoic acid; C23:0, Tricosanic acid; C24:0, Lignoceric acid; C14:1, Myristoleic acid; C16:1, Palmitoleic acid; C17:1, Heptadecenoic acid; C18:1n9t, Elaidic acid; C18:1n9c, Oleic acid; C18:2n6t, Linolelaidic acid; C18:2n6c, Linoleic acid; C18:3n3, Linolenic acid; C18:3n6, γ-Linolenic acid; C20:1n9, Eicosenoic acid; C20:2, Eicosadienoic acid; C20:3n6, Cis-8,11,14-Eicosatrienoate; C20:3n3, Cis-11,14,17-Eicosatrienoate; C20:5N3, Timnodonic acid; C22:1n9, Erucic acid; C22:2, Docosadienoic acid; C22:6n3, Docosahexaenoic acid; C24:1n9, Nervonic acid; MUFA, monounsaturated fatty acid; PUFA, polyunsaturated fatty acid;*

# *difference between CN group and BS group in breast muscle;*

† *difference between CN group and BS group in thigh muscle;*

‡ *difference between breast muscle and thigh muscle; p < 0.05 were taken to indicate statistical significance*.

## Discussion

In the present study, we mainly investigated the effects of dietary supplementation with *B. subtilis* DSM 32315 on carcass traits, meat quality, AA, and fatty acid profile of broiler chickens. The results of the present study showed that dietary supplementation with *B. subtilis* DSM 32315 has a remarkable effect on carcass traits and could improve the meat quality and meat flavor of broiler chickens through improving the AA and fatty acid profiles. Carcass traits provide important indices in broiler production. In the present study, dietary supplementation with *B. subtilis* significantly increased the breast muscle and significantly decreased the abdominal fat of broiler chickens. However, *B. subtilis* supplementation had no effect on the carcass weight, dressing percentage, semi-eviscerated percentage, eviscerated percentage, and leg muscle, which is consistent with the results from Yadav et al. (31) who reported that dietary supplementation with *B. subtilis* (DM 03, TAM 4 and IQB 350) significantly increased the breast muscle weight of broilers. In contrast, Cramer et al. (11) reported that dietary supplementation with *B. subtilis* did not affect the breast muscle weight of broilers exposed to chronic heat stress, and Park and Kim (13) reported that dietary supplementation with *B. subtilis* RX7 or B2A did not affect the abdominal fat of broilers challenged with *Salmonella typhimurium*. This is probably because the beneficial effects of *B. subtilis* in broilers were markedly strain-dependent (7, 32). Abdominal fat is an important index used to measure lipid deposition in broilers (33, 34). Excessive fat deposition is undesirable, because it degrades meat quality, decreases feed efficiency, and increases production and health costs (35, 36). The lower abdominal fat in broiler chickens supplemented with *B. subtilis* in the present study indicated that *B. subtilis* can lead to a higher economic efficiency since the public tends to favor low-fat-content chicken.

The pH value, meat color (lightness, redness, and yellowness), drip loss, cooking loss, and shear force are commonly used indices for evaluating meat quality (37). Previous studies have reported that dietary supplementation with *B. subtilis* could improve the carcass traits and meat quality of broilers by improving the pH, tenderness, and color (11–13). pH is related to shelf life, color, and water-holding capacity of the meat, which are determined mostly by postmortem conversion of muscle glycogen to lactic acid, and often used as an important indicator to evaluate meat quality (38, 39). The higher pH_24h_ in the muscle of broilers fed with *B. subtilis* indicated a better shelf life, color, and water-holding capacity of meat. Similarly, Cramer et al. (11) also showed that dietary supplementation with *B. subtilis* increased the pH_24h_ of breast muscle. In contrast, Park and Kim (13) and Bai et al. (12) reported that dietary supplementation with *B. subtilis* had no effect on the pH value of meat. *B. subtilis* supplementation did not affect the meat color (a^*^, b^*^, and L^*^) in the present study, which is consistent with Jeong and Kim (21) and Cramer et al. (11), who also reported that *B. subtilis* had no effect on the meat color. However, in contrast, Bai et al. (12) reported that *B. subtilis* could decrease L^*^ and b^*^, and increase a^*^. Drip loss and cooking loss could reflect the water-holding capacity and are related to nutrition, flavor, and juiciness, because some nutrients are easily lost during exudation by water loss (40, 41). In the present study, dietary supplementation with *B. subtilis* decreased the drop loss and cooking loss of the breast muscle after storage for 1 day, which suggested the increase of water-holding capacity of meat. The results of our study support the previous research, which concluded that dietary supplementation with *B. subtilis* RX7 or B2A in broiler diets increased the drip loss of breast meat (13). However, the detailed reasons for *B. subtilis* decreased the drip loss and cooking loss was unclear. Tenderness is considered as the most important trait for consumers, which can be represented by shear force (42). It was clear from our study that the administration of *B. subtilis* had a beneficial effect on meat tenderness with a decreased shear force. Inconsistent results between studies have been reported for the impact of *B. subtilis* supplementation on meat tenderness. Similarly to the present study, Bai et al. (43) reported that the administration of *B. subtilis* in the diets could decrease the shear force and thus improve muscle tenderness, whereas Cramer et al. (11) and Abdulla et al. (44) had found that dietary supplementation with *B. subtilis* had no difference in shear force. Therefore, the present study indicated that dietary supplementation with *B. subtilis* could improve the meat quality of broilers by improving the pH and tenderness and by decreasing the drop loss and cooking loss.

In the current study, *B. subtilis* treatment did not affect the routine nutrients (moisture, crude protein, ether extract, and crude ash) in both breast and thigh muscles compared to the control group, but it showed that dietary supplementation with *B. subtilis* had a tendency to improve the ether extract content in the thigh muscle. Similarly, Zhou et al. (16) indicated that dietary probiotic *Bacillus coagulans* supplementation did not affect moisture, crude protein, ether extract, and crude ash content in the muscles of Guangxi Yellow chicken. In contrast, Abdulla et al. (44) showed that dietary supplementation with *B. subtilis* significantly decreased the fat content in breast and thigh muscles of broilers. Intramuscular fat content is an important sensory characteristic of meats and is related to tenderness, succulence, and flavor of meats (45). The higher fat content of thigh muscle from *B. subtilis* treatment in the present study suggested better tenderness, succulence, and flavor of meats.

The AA content in meat is important to evaluate the quality and flavor of meat. EAA determines the muscle protein quality, and flavor amino acid (FAA), such as alanine (Ala), glycine (Gly), Glu, aspartic acid (Asp), and serine (Ser), especially Glu, can greatly contribute to the taste of meat (38, 46). In the breast muscle, Met, Lys, Glu, total EAA, and total AA were increased by *B. subtilis* treatment in the present study. In the thigh muscle, only Glu content was increased by *B. subtilis* treatment. The reasons for this phenomenon might be due to the higher content of total AA in the breast muscle that made it more sensitive to FAAs. The results from Guo et al. (37) and Chen et al. (47) also support this opinion, who reported that breast muscle contained higher levels of total AAs than those of thigh muscle in broiler chickens. Lys and Met are two kinds of important EAA in meat of broilers. The higher Lys and Met in the meat of broilers fed with *B. subtilis* meant that *B. subtilis* had a beneficial effect on improving the protein quality and nutrition value of muscle. Glu is one of the most important FAA, which is the primary flavor molecule and functions in meat freshness and buffering salty and sour tastes (48). The Glu content in breast muscle and thigh muscle both increased due to *B. subtilis* treatment, indicating that *B. subtilis* has a role in promoting the meat flavor. In a word, *B. subtilis* could improve the meat protein quality as well as meat flavor of broilers.

It is well-known that fatty acids, including MUFA, polyunsaturated fatty acids (PUFA), and SFA, are an important indicator to evaluate the quality and nutritional value of meat and they also are the basis for the characteristic flavor of meat (49). The major fatty acids in chicken meat are C16:0, stearic acid (C18:0), C18:1n9c, and linoleic acid (C18:2n6c) (50). It is generally accepted that higher dietary intakes of SFA, especially C16:0 and myristic acid (C14:0), are positively correlated with increased risk of coronary heart disease, due to their ability to raise the total cholesterol and low-density lipoprotein (51, 52). In the present study, *B. subtilis* treatment significantly decreased C14:0, C16:0, and C20:0 in the breast muscle, and significantly decreased C16:0 and total SFA in the thigh muscle, which indicated that the muscles from broilers treated with *B. subtilis* might be more beneficial to human health. The UFA not only is the precursor substance to meat flavor, but also plays a wide range of roles in promoting growth, scavenging free radicals, and regulating lipid metabolism (49). In the present study, *B. subtilis* treatment significantly increased C16:1, C18:1n9c, C20:3n6, and total MUFA in the breast muscle, and significantly increased C16:1, C18:1n9c, and total MUFA in the thigh muscle. C18:1n9c is the most abundant UFA, which is suggested to be positively associated with the softness of fat and the sensory quality of meat; thus, rich oleic acid content can make meat tasty (51, 52). The higher C18:1n9c content in muscles from broilers treated with *B. subtilis* meant a better meat quality. C18:2n6c was reported to cause a negative flavor in meat (53). *B. subtilis* treatment had no effect on C18:2n6c content in both breast and thigh muscles, which meant that there was no adverse effect on meat flavor when broilers are supplemented with *B. subtilis*. In summary, *B. subtilis* could improve the composition of muscle fatty acids by decreasing the SFA content and increasing the UFA content, thus improving the meat quality as well as meat flavor.

## Conclusions

In conclusion, dietary supplementation with *B. subtilis* could improve the carcass traits and meat quality of broilers by improving the pH and tenderness, decreasing the drip loss and cooking loss, and adjusting the composition of AAs and fatty acids.

## Data Availability Statement

The original contributions presented in the study are included in the article/Supplementary Material, further inquiries can be directed to the corresponding author/s.

## Ethics Statement

The experimental procedures involving animals were reviewed and approved by the Animal Welfare Committee of the Guizhou Normal University (Guiyang, China).

## Author Contributions

XT and HL: conceptualization, investigation, resources, and methodology. XT and XL: formal analysis and data curation. XT: writing—original draft preparation, writing—review and editing, project administration, funding acquisition, and supervision. All authors have read and agreed to the published version of the manuscript.

## Funding

This research was supported by the China Overseas Expertise Introduction Program for Discipline Innovation (No. D17016),the Key Project of Science and Technology Program of Guizhou Province (No. 5411 2017 Qiankehe Pingtai Rencai), the World Top Discipline Program of Guizhou Province (No. 125 2019 Qianjiao Keyan Fa), the Natural Science Research Project of Education Department of Guizhou Province[Qianjiaohe KY Zi (2021) 294], and the Doctoral Launched Scientific Research Program of Guizhou Normal University [GZNUD (2018) 26].

## Conflict of Interest

The authors declare that the research was conducted in the absence of any commercial or financial relationships that could be construed as a potential conflict of interest.

## Publisher's Note

All claims expressed in this article are solely those of the authors and do not necessarily represent those of their affiliated organizations, or those of the publisher, the editors and the reviewers. Any product that may be evaluated in this article, or claim that may be made by its manufacturer, is not guaranteed or endorsed by the publisher.
